# Mental State Understanding in Children with Agenesis of the Corpus Callosum

**DOI:** 10.3389/fpsyg.2017.00094

**Published:** 2017-02-06

**Authors:** Beatrix Lábadi, Anna M. Beke

**Affiliations:** ^1^Department of General and Evolutionary Psychology, Institute of Psychology, University of PécsPécs, Hungary; ^2^Obstetric and Gynecology Clinic No. 1, Semmelweis UniversityBudapest, Hungary

**Keywords:** agenesis of the corpus callosum, mentalizing ability, emotion recognition, executive function, behavioral problems

## Abstract

Impaired social functioning is a well-known outcome of individuals with agenesis of the corpus callosum. Social deficits in nonliteral language comprehension, humor, social reasoning, and recognition of facial expression have all been documented in adults with agenesis of the corpus callosum. In the present study, we examined the emotional and mentalizing deficits that contributing to the social-cognitive development in children with isolated corpus callosum agenesia, including emotion recognition, theory of mind, executive function, working memory, and behavioral impairments as assessed by the parents. The study involved children between the age of 6 and 8 years along with typically developing children who were matched by IQ, age, gender, education, and caregiver's education. The findings indicated that children with agenesis of the corpus callosum exhibited mild impairments in all social factors (recognizing emotions, understanding theory of mind), and showed more behavioral problems than control children. Taken together, these findings suggest that reduced callosal connectivity may contribute to the development of higher-order social-cognitive deficits, involving limits of complex and rapidly occurring social information to be processed. The studies of AgCC shed lights of the role of structural connectivity across the hemispheres in neurodevelopmental disorders.

## Introduction

Agenesis of the corpus callosum (AgCC) is a common cerebral malformation resulting from a failure to develop fibers that provide the largest connective tract between the two hemispheres. The corpus callosum consists of over 200 million axons that transfer information between the two hemispheres. Callosal anomalies are the most frequent malformations in the brain, with imaging studies indicating that AgCC occurs in 1:4000 live births (Wang et al., 2004; Glass et al., 2008), and 3–5% of neurodevelopmental disorders involve callosal malformation (Bodensteiner et al., 1994). The developmental absence (agenesis) of the corpus callosum can occur in a variety of conditions that disrupt the early development of the callosal fibers. Current studies suggest that callosal dysgenesis can be reflected in inborn errors of metabolism, chromosomal anomalies, or genetic syndromes (Bedeschi et al., 2006). AgCC can encompass either total or partial absence of the corpus callous, as well as hypoplasia (formation of a thinner than expected corpus callosum). Surprisingly, the comparison of partial and total agenesis of the corpus callosum showed only slight differences in medical and behavioral outcomes (Paul et al., 2007). Patients with the syndromic form of corpus callosum dysgenesis (a callosal abnormality associated with other genetic syndromes e.g., Aicardi syndrome) show severe developmental delay and intellectual disabilities (Sztriha, 2005). Whereas individuals with isolated AgCC (Symington et al., 2010), meaning they do not have additional syndromes or disorders or evidence of other brain pathology, typically only have mild behavioral and cognitive problems (Moutard et al., 2003). However, the outcome of isolated AgCC is often unclear because their intellectual development can range from severely delayed to “perfectly normal” (Paul et al., 2007).

Initial studies examining individuals diagnosed with AgCC suggested impairments in their higher-order cognitive functions and social interaction. Neuropsychological studies found evidence for cognitive impairments in abstract reasoning (Brown and Paul, 2000), problem solving, and processing speed (Marco et al., 2012). These cognitive abilities become more impaired as the task's complexity increases (Brown and Paul, 2000). However, those with isolated AgCC do not show severe general cognitive disabilities (Sauerwein et al., 1994) or language impairments regarding naming, receptive language, and lexical reading abilities (Brown and Paul, 2000). While deficits were observed in linguistic pragmatics, AgCC sufferers have difficulty understanding idioms, proverbs (Banich and Brown, 2000), and narrative humor (Paul et al., 2003) as they tend to ignore the second-order meaning of narratives or conversations.

Acallosal patients generally exhibit difficulties in social cognition and social behavior, with adults showing impairment in understanding others' mental states (Symington et al., 2010) and in recognizing emotions (Bridgman et al., 2014). The deficit in emotion recognition seems to be directly associated with atypical facial scanning; adults with AgCC spend less time in the eye region while observing emotional expressions of others (Bridgman et al., 2014), resulting in poorer detection of others' emotional and mental states (Baron-Cohen et al., 1997). The social-cognitive impairment in AgCC was also demonstrated by mild theory of mind (ToM) deficits (Symington et al., 2010), poor social self-awareness (Brown and Paul, 2000), and difficulties in social perspective taking (Symington et al., 2010; Turk et al., 2010). Overall, the findings of social cognition research suggest that AgCC patients have particular difficulties understanding complex socio-emotional and life-like contexts of everyday situations. These social cognitive impairments in people with callosal agenesis overlap with the profile of autism spectrum disorders (ASD). Individuals with ASD show similar patterns of emotion recognition, being significantly worse at recognizing emotions compared with normal controls, particularly when only the eye region of faces is presented. There is also evidence that AgCC individuals share the characteristic of impaired social cognition with ASD patients, especially with respect to the difficulties in recognizing another person's mental states, feelings, intentions, and goals. Survey studies, completed by caregivers of children with AgCC, reported that a significant number of children and adults have problems with social behaviors (Badaruddin et al., 2007), and exhibit significant autistic symptomatology (Moes et al., 2009; Lau et al., 2013). To clarify the relationship between the autistic-like behavior and callosal agenesis, a recent study (Paul et al., 2014) directly compared AgCC adults with ASD adults. Using the Autism Diagnostic Observation Schedule, they found that one third of the adults with AgCC met the clinical criteria for autism, whereas very few subjects were consistent with the ASD diagnosis when developmental history was included. The autistic traits seen in AgCC patients appear to emerge in differing time-courses, depending on the age of the AgCC patient.

Despite the convergent evidence reviewed here, with regard to social and cognitive deficits in AgCC, much work is still needed. At present there are only a few studies that have directly examined the mentalizing abilities in persons with agenesis of the corpus callosum, and these have mainly been case studies. In addition, even less research has been conducted in children with AgCC that has specifically investigated the developmental course of social-cognitive domains, including emotion recognition and theory of mind. Previous studies involving adults implicitly proposed that these impairments are not likely to be exhibited in younger children with AgCC (Paul et al., 2014), because normally, the corpus callosum is not yet fully myelinated until adolescence (Giedd et al., 1996). However, parent reported assessment studies have suggested that children with AgCC are more likely to exhibit autistic symptoms, including social-cognitive deficits, compared with adults (Moes et al., 2009). Additionally, the specific social and communicative abnormalities emerge early on, at about the age of three, in ASD children who share important clinical and neuroanatomical parallels with AgCC children. Therefore, the social and cognitive deficits in emotion recognition and mentalizing capacities are more likely to occur in childhood in AgCC children. The present study addresses this issue in a sample of 6–8 year old children with isolated AgCC. We chose this age range because the social and higher-order cognitive functions (theory of mind, understanding emotions, inhibitory control, working memory), which are necessary for school readiness, are available for typically developing children at this age.

The first aim of this study was to characterize the social and higher-order cognitive functions in a sample of 18 children diagnosed with isolated AgCC. In light of previous studies examining social and cognitive functions in AgCC and ASD individuals, we predicted that when given the task of recognizing complex mental states from faces, children would perform poorly; but would perform normally at recognizing basic emotions. Additionally, we expected that AgCC children would have more difficulty identifying mental states or emotions that involved the eye region. We also hypothesized that children with AgCC would have difficulties in inferring the mental states of others, but this deficiency only becomes apparent in more complex situations, when more information must be processed and integrated. We expected that children would be more likely to pass first-order false belief tasks, but would perform poorly in second-order false belief tasks. Alternatively, the impaired social cognitive function could reflect deficits in inhibitory control and working memory. Here, we predicted that performance of inhibitory control becomes more impaired in children with AgCC relative to normally developing children, and the impaired executive functions makes a unique contribution to the ability of theory of mind. In this study, we test the relative contributions of inhibitory control, working memory, and assessment of intelligence to AgCC children's social abilities (ToM and emotion - mental state recognition). Our second aim was to examine the relationship between social cognition and the severity of behavioral problems in children with and without corpus callous agenesia. To answer these research questions, we employed validated social cognitive tasks (emotion recognition, theory of mind, executive function, and working memory), and parent-reported assessments. Our study is the first comprehensive direct comparison of AgCC children with typically developing children, in cognitive and social domains.

## Materials and methods

### Participants

Participants included 18 children with isolated corpus callosum agenesia between the age of 6 and 8 years, and 18 normally developing children as control (Table 1 shows the demographic and psychological background information). Groups were matched with respect to IQ, age, gender, children's education, and caregiver's education. The two groups had exactly the same number of males (14) and females (4), and they were perfectly matched for age and education level; each child with AgCC was individually paired to a typically developing child. In the AgCC cohort, five were left-handed and four were ambidextrous, while in the control group two were left-handed and 16 were right-handed, with handedness being determined by the administration of the Edinburgh Handedness Inventory. The AgCC group included 16 with complete agenesis of the corpus callosum and two with partial agenesis (we did not exclude two children with partial AgCC because the individual connectivity pattern and differences was beyond the scope of our study, and previous studies also included both partial and complete AgCC individuals, e.g., Lau et al., 2013; Paul et al., 2014). The inclusion criteria for both groups were: 6–8 years of age, IQ scores >75, no major head trauma or neurosurgery, no additional genetic syndromes (e.g., Aicardi syndrome), and no severe psychopathology (children with anxiety, ADHD, and children undergoing psychotherapy treatment and/or taking psychotropic medication were excluded). Regular and neuropsychological examinations for all AgCC participants were conducted at the Neurology Department of Obstetric and Gynecology Clinic (Semmelweis University) in Budapest. The controls were recruited from the local kindergarten and primary school.

**Table 1:** **Demographic and background psychological measures**.

	**AgCC (*N* = 18)**	**Control (*N* = 18)**	***t, x^2^ p*-value**
**AGE (YEARS)**			
Mean (SD)	6.80 (0.84)	6.93 (0.76)	*t* = −0.496 (*p* = 0.623)
Range	5.9–8.1	6.1–8.0	
**FSIQ**			
Mean (SD)	98.16 (8.79)	100.83 (6.48)	*t* = −1.036 (*p* = 0.308)
Range	85–108	88–109	
**PIQ**			
Mean (SD)	94.66 (8.79)	98.33 (8.12)	*t* = −1.081 (*p* = 0.287)
Range			
**VIQ**			
Mean (SD)	96.94 (14.00)	102.83 (7.74)	*t* = −1.561 (*p* = 0.128)
Range			
**GENDER**	**M: 14 F: 4**	**M: 14 F: 4**	***x*^2^ = 0.0, (*p* = 1.0)**
**HANDEDNESS**	**L: 5 R: 9 A:4**	**L: 2 R: 16**	***x*^2^ = 7.246, (*p* = 0.02)**
**CHILDREN**	**PRESCHOOL:**	**PRESCHOOL:**	***x*^2^ = 0.0, (*p* = 1.0)**
**EDUCATION**	**10 SCHOOL: 8**	**10 SCHOOL: 8**	
**CAREGIVER EDUCATION**			
Mean (SD)	12.77 (2.36)	13.00 (2.11)	*t* = −0.297 (*p* = 0.786)
Range	11.0–17.0	11.0–17.0	

*The comparison based on the t**-**test for age, FSIQ, PIQ, VIQ, caregiver education and x^2^ for gender, handedness and children education*.

Children with AgCC were first diagnosed before birth; the absence of the corpus callous in utero was identified upon routine high-resolution ultrasound, and, then a magnetic resonance imaging (MRI) scan confirmed the diagnosis. For all participants with AgCC, previous MRI and radiological reports were gathered to confirm the diagnosis of complete or partial AgCC by an independent second neuroradiologist. Images were evaluated for the presence and size of the corpus callosum, Probst bundle, anterior commissure, white matter abnormalities, and cortical malformations (e.g., subcortical heterotopia, polymicrogyria). Participants with AgCC were included if they had structural findings that commonly co-occur with AgCC: Probst Bundle, colpocephaly, and interhemispheric cysts. Children with other structural brain abnormalities (known genetic syndrome, frontal lobe dysgenesis) were excluded. The presence of anterior commissure was confirmed in all participants. The intelligence scores, based on the Test of Wechsler Intelligence Scale for Children - III (Hungarian standard version), were also collected from previous neuropsychological records (assessed within a year). Control participants' intelligence scores were also established using the Test of Wechsler Intelligence Scale for Children -III.

The caregiver of each participant signed an informed consent. All participants were treated in accordance with the Hungarian Psychological Association Ethical Codes. This study was carried out in accordance with the recommendations of Psychology Research Guidelines of the Ethical Committee of the Hungarian Psychological Association, with written informed consent from each caregiver of the subjects, in accordance with the Declaration of Helsinki.

### Measures

#### Theory of mind

To assess ToM, we used two classic False Belief Tasks with some modification to test AgCC children's ability to understand others' mind. The first-order false belief task was the traditional *The Smarties tube test* (Perner et al., 1989). The task involves a familiar Smarties box, but filled with pencils instead of candies. The experimenter first asks the child “*What do you think is in this box?,”* and the child naturally replies “*Candies,”* because they have an expectation of what is in the box (false belief). The child is then shown the pencils inside the box. Then the experimenter closes the lid of the box and asks the child two belief questions. The first question is “*When I first showed you this box what did you think was inside?,”* and the second question is “*What will your mother (who did not see the pencils) think is inside the box?”* If the child has a theory of mind, they will realize that their mother would also think candies are inside, and a normal 4-year-old child mostly answers “*Candies,”* by referring the other's false belief, but 3-year-old children or children with impaired ToM usually reply “*pencils.”*

The second-order false belief task was a modification of the Sally-Anne test (Baron-Cohen et al., 1999). In the original task (Baron-Cohen et al., 1985) the child is introduced to two dolls, Sally and Anne, who are playing with a marble. The dolls put the marble in a basket then Sally leaves the scene. Anne takes the marble out of the basket and she puts it away in a different container. When Sally returns the child is asked “*Where will Sally search for the marble?”* The child fails the theory of mind task if she answers that Sally will search for it in the second container. The second-order modification of the Sally-Anne task is that when Sally leaves, she looks back through the key-hole while Anne is transferring the marble to the new location. When Sally returns, the test question is no longer “*Where will Sally search for the marble?,”* instead it is “*Where does Anne think Sally will search for the marble?”* We used this modification of the Sally-Anne Task to test the children's second-order theory of mind ability. Children were successful if they responded correctly to both the test and control questions. These tasks were scored as pass = 1, fail = 0. Performance across the two tasks was summed (range 0–2) to create a single indicator of false belief understanding. Additionally, we also analyzed each test individually as the indicator of first-order false belief test and second-order false belief test.

#### Emotion and mental state recognition

We administered the Faces Test (Baron-Cohen et al., 1997) to measure the emotion and mental state recognition of children. The Faces Test consists of 20 photographs of an actress posing, 10 photos of basic emotions (happy, sad, angry, afraid, disgust, distress, surprise), and 10 photos of complex mental states (scheme, guilt, admire, interest, thoughtfulness, quizzical, bored, arrogant, flirting, quizzical). In our experiment, under each photo, two words were typed, but only one described the target basic emotion or mental state the actress was posing. Subjects were presented with 20 photos (10 basic emotions and 10 complex mental states) separately in a random order. For each photo, the experimenter read the two words under the photo, and the child was asked to choose the emotion/mental state that best described what the person was thinking or feeling in the picture. Each trial was scored as pass = 1, fail = 0. The dependent measure was the number of correct answers for basic emotions and for complex mental states. Performance across the two conditions was summed (range 0–20) to create a single indicator of emotion and mental state recognition.

#### Executive function

Two executive function tasks were administered, providing measures of inhibitory control (Day and Night Stroop), and working memory (Digit Span forward and backward).

For working memory, we administered the standard Wechsler Digit Span Task to measure the working memory capacity. This test requires the examiner to verbally present digits at a rate of one per second, and children are asked to repeat the digits verbatim in the same order (forward test). The backward test requires the participant to repeat the digits in reverse order. The number of digits increases by one until the participant consecutively fails two trials of the same digit span length. The task was preceded by a brief training procedure. Two practice items preceded the experimental trials, and the task was only started if the child passed the practice trials. Children were administered two different trials of each sequence length, which ranged from two to nine.

To measure inhibitory control, the Day and Night Stroop task (Gerstadt et al., 1994) was used to assess executive function measurement of interference control in young children. Children were instructed to say the word “night” when they saw a white sun card and to say “day” when shown a black moon card. We used two conditions, an Incongruent Condition to test the ability of inhibition and a Congruent Condition as a control. In the Incongruent Condition, children were required to say the opposite of what was shown on the day-night cards, maintain the task instructions over the procedure, and inhibit a dominant response associated directly to the stimulus while executing the subdominant response. In the Congruent condition, children simply said what the stimulus represented. The order of the conditions was counterbalanced across participants, for half of the participants, the experiment started with the presentation of Congruent Condition, while for the other half of the participants the Incongruent Condition was conducted. The participant did four practice trials. In each condition 16 trials were administered, in which eight night cards and eight day cards were shown in a pseudo-random order (n(ight), d(ay), n, d, d, n, d, n, n, d, d, n, d, n, n, d, n, d). No feedback was given to the children. The task was presented on a computer screen and was controlled by PsychoPy, presenting the stimuli and recording the participants' responses. The dependent measure was the total number of correct answers for each condition. Response latency was not measured because most of the children were unable to correctly use the response panel.

#### Behavior questionnaire

We used the validated Hungarian version of the extended Strength and Difficulty Questionnaire (Goodman, 1997, 1999, SDQ) to measure the children's emotional and behavioral difficulties. The SDQ was administered by parents and covers the major areas of emotional and behavioral difficulties, and predicts psychiatric disorders. The SDQ consists of 25 items, divided into five subscales: the prosocial subscale, the inattention-hyperactivity subscale, the emotional symptoms subscale, the conduct problems subscale, and the peer problems subscale. Each item can be scored “not true,” “somewhat true,” or “certainly true.” The extended version includes questions that ask whether the respondent considers the young person to have a problem and its impact on their social emotional life. All subscales expecting pro-social behavior are summed to compute a total difficulties score. The dependent variables taken from the SDQ include the total score of difficulties and five subscale scores. The SDQ is available on the internet website: www.sdqinfo.com.

### Procedure

Subjects were tested individually in a quiet room, at the clinic of Semmelweis University for AgCC children, and at the local primary school for control children. All children were tested in a single session for the target tasks, the control children completed the intelligence test in a separate session. Intelligence was assessed in the AgCC children prior to the study, by a neuropsychologist during a regular yearly visit. On arrival, the child was asked to be seated at the table, then the experimenter explained that they were going to play some “games.” Prior to each test, participants were trained on how to do the task. Parents received a child behavior questionnaire and were asked to complete the questionnaires and return them. All children were administered individually over two separate sessions, and there was no time limit.

### Data analysis

All statistical analyses were conducted using IMB SPSS Statistics (Version 22.0). We used an independent *t*-test and chi^2^ test to examine the group differences between the AgCC group and control group for social-cognitive factors and behavioral problems. We then computed correlations (Pearson's *r*) to test our prediction that social-cognitive abilities would be associated with behavioral symptoms. We used a significance level of 0.05 (two-tailed) for all tests.

## Results

### Theory of mind

First, we evaluated the control questions (reality and memory) of the false belief tasks, and only those subjects who passed the control questions were included in the present analysis. One AgCC child was excluded from the analysis due to failing the control questions. We compared the performance of AgCC children with control children for on each false belief test using *chi*^2^ test. The proportion of subjects in the AgCC group who passed either the first-order false belief task [Smarties tube test, *x*^2^_(35)_ = 7.098, 1*df*, *p* = 0.00], or the second-order false belief task [modified Sally-Anne false belief task, *x*^2^_(35)_ = 3.736, 1*df, p* = 0.05] was significantly smaller than that of the control group. Finally, the Mann-Whitney non-parametric test confirmed that children with AgCC performed poorer, with lower total scores on the ToM tasks [*z*_(35)_ = −2.612, *p* = 0.009]. Table 2 shows numbers of children who passed the Smarties or Sally-Anne M false belief tasks.

**Table 2:** **Number of subjects in each group who passed on Smarties or Sally Anne M belief tasks (an AgCC child did not pass the control questions)**.

	**AgCC (*N* = 17)**	**Control (*N* = 18)**	**Total (*N* = 35)**
Smarties	8	16	24
Sally Anne M	4	10	14

Additional analysis of covariance (ANCOVA) was conducted in order to investigate the effect of verbal intelligence on false belief performance. When VIQ was applied as a covariate in ANCOVA, it indicated that false belief scores were significantly poorer for the AgCC group [*F*_(1, 32)_ = 6.42, *p* = 0.01, η*p*^2^ = 0.01]. ANCOVA shows that the main effect of verbal ability (VIQ) on false belief performance was not significant [*F*_(1, 32)_ = 2.90, *p* = 0.09, η*p*^2^ = 0.08].

### Emotion and mental state recognition

**Figure 1:**
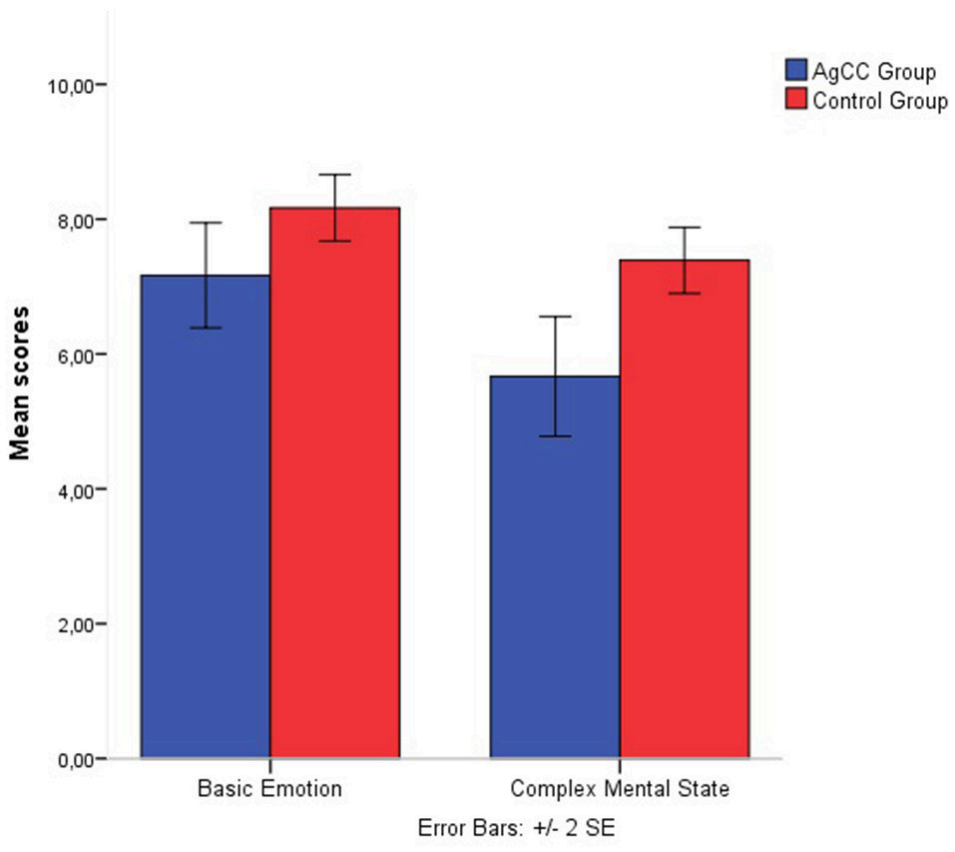
**Emotion recognition and mental state recognition mean accuracy by group**. Error bars represent standard error.

First, we compared the performance on recognition of basic emotions and complex mental states in AgCC and control subjects. The AgCC group were less accurate than the control group on overall Faces scores [for total scores *t*_(35)_ = −3.483, *p* = 0.001, *d* = 1.16]. Subjects with AgCC showed poorer performance (*M* = 12.83, *SD* = 3.05) on selecting the target emotional and mental states compared with the control children (*M* = 15.55, *SD* = 1.29). Repeated measures of ANOVA, comparing group (AgCC vs. control) and complexity of mental states factor (basic vs. complex), revealed a significant group effect [*F*_(1, 34)_ = 12.13, *p* = 0.001, η*p*^2^ = 0.26], indicating that children with AgCC were less accurate than control subjects. A significant difference was also found for complexity of mental states factor [*F*_(1, 34)_ = 15.93, *p* = 0.00, η*p*^2^ = 0.31]; children recognized the basic emotional mental states more accurately than complex mental states in both groups. The group × complexity interaction was not significant [*F*_(1, 34)_ = 1.602, *p* = 0.21, η*p*^2^ = 0.045] for complexity and group factors. The mean scores (Figure 1) indicate that subjects with AgCC performed less accurately on both basic emotion trials (*M* = 7.16, *SD* = 1.65) and complex mental state recognition trials (*M* = 5.66, *SD* = 1.87) compared with the control children (for basic emotion: *M* = 8.16, *SD* = 1.04, and for complex mental states: *M* = 7.38, *SD* = 1.29).

Table 3 shows the number of children choosing the correct basic emotion or complex mental state for each trial. Using *chi*^2^ test to compare the performance of AgCC individuals and control subjects on each trial, the analysis revealed that there was a significant difference between the AgCC group and control group, but only on the “angry vs. afraid” and “sad vs. disgust” trials in the basic emotion trials. As for the trials of the complex mental states, children with AgCC were significantly less accurate on the trials of the “guilt vs. arrogant,” “interest vs. disinterest,” “scheming vs. arrogant” compared with control subjects (Table 3).

**Table 3:** **Results of FACES Test, showing number of subjects passing each trial**.

	**AgCC subjects (*N* = 18)**	**Control subjects (*N* = 18)**	**Pearson *x^2^***
**BASIC EMOTIONS**			
Picture 1: Happy vs. Surprise	18	18	0.0
Picture 2: Afraid vs. Angry	13	17	3.200
Picture 3: Surprise vs. Happy	17	18	1.029
Picture 4: Disgust vs. Sad	14	18	4.500^*^
Picture 5: Sad vs. Disgust	15	17	1.125
Picture 6: Angry vs. Afraid	10	9	0,111
Picture 7: Surprise vs. Happy	9	9	0.0
Picture 8: Distress vs. Sad	11	10	1.114
Picture 9: Happy vs. Surprise	15	16	0.232
Picture 10: Angry vs. Afraid	7	15	7.481^**^
**COMPLEX MENTAL STATES**			
Picture 11: Scheming vs. Arrogant	5	12	5.461^*^
Picture 12: Guilt vs. Arrogant	10	16	4.985^*^
Picture 13: Thoughtful vs. Arrogant	13	17	3.200
Picture 14: Admiring vs. Surprise	15	16	0.232
Picture 15: Quizzical vs. Guilt	11	10	0.114
Picture 16: Playful vs. Happy	13	11	0.500
Picture 17: Bored vs. Sleepy	10	11	0.114
Picture 18: Interested vs. Disinterested	9	16	6.415^*^
Picture 19: Interested vs. Disinterested	5	12	5.461^*^
Picture 20: Arrogant vs. Guilt	11	11	0.0

*The first term is the target (correct) term. The position of the target term and the order of the picture pairs were randomized*.

** *p < 0.01*,

* *p < 0.05*.

### Executive function

#### Working memory

The independent sample *t-*test results showed no significant effect for the forward digit span (*p* = ns.), or for the backward digit span (*p* = ns.).

#### Inhibitory control

**Figure 2:**
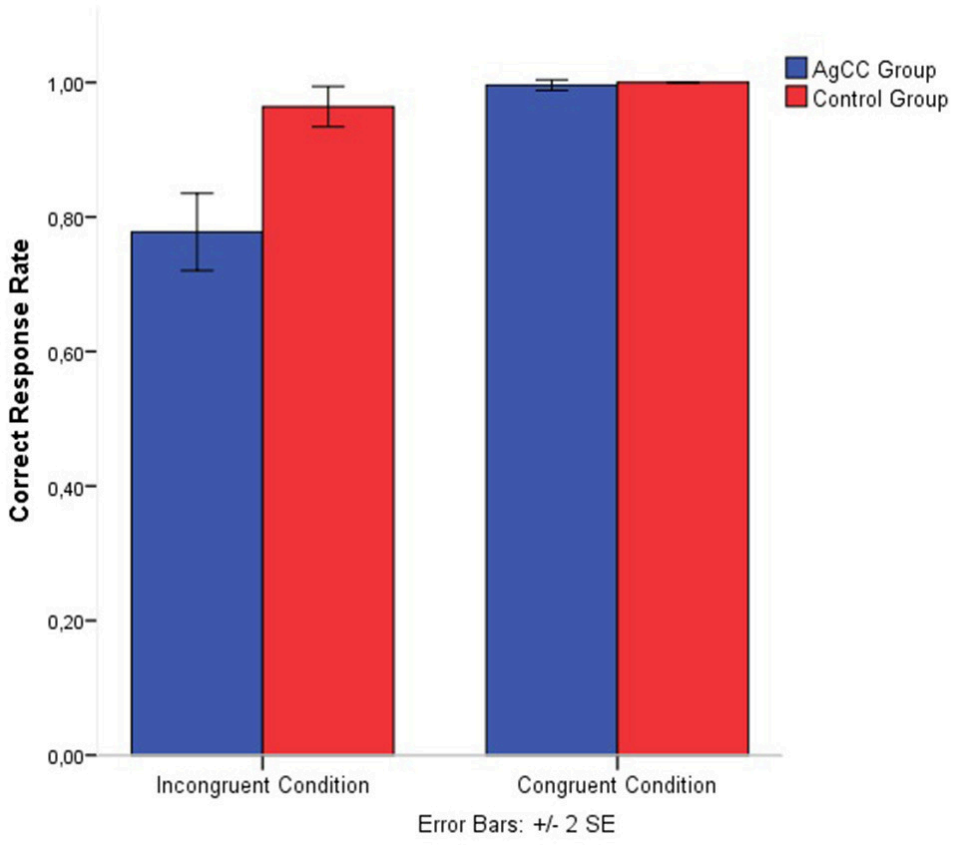
**Day and night Strop accuracy performance separated by group and congruency**. Error bars represent standard error.

A 2 groups (AgCC vs. control) × 2 conditions (congruent vs. incongruent) mixed model ANOVA was conducted for performance (correct response rate). The analysis of performance showed a significant main effect for the condition [*F*_(1, 30)_ = 65.29, *p* = 0.000, η*p*^2^ = 0.684], and for the group [*F*_(1, 30)_ = 36.39, *p* = 0.000, η*p*^2^ = 0.548], and for the interaction between group and condition [*F*_(1, 30)_ = 33.61, *p* = 0.000, η*p*^2^ = 0.527]. The results demonstrate that Stroop-like interference is higher in children with AgCC (*M* = 14.0, *SD* = 0.41) compared with control children (*M* = 17.35, *SD* = 0.46), regarding the performance (Figure 2).

### Relationship between social and cognitive factors

We computed additional correlational analysis for both groups separately to determine correlations between social and cognitive factors (Table 4). Within the AgCC group, there was a medium strength correlation between forward digit span and basic emotion recognition, as well as between backward digit span and complex mental state recognition and inhibitory controls, with significant correlation coefficients ranging from *r* = 0.41 to *r* = 0.62, *p* < 0.05. For the inhibitory control measures, there was no significant correlation with social factors (false belief and emotion and mental state recognition). Similarly, intelligence factors (general IQ, verbal IQ, and non-verbal IQ) also showed no significant correlation with social and cognitive factors (theory of mind, emotions, and mental state recognition, inhibitory control and working memory). For the control group, significant correlations were only found between backward digit span and false belief [*r*_(18)_ = 0.51, *p* = 0.03].

**Table 4:** **Correlation *r* between social factors and behavioral problems measures separately for each group (AgCC Group/Control Group)**.

**Control**	**AgCC**							
		**1**.	**2**.	**3**.	**4**.	**5**.	**6**.	**7**.
	1. False belief	1	0.22	0.49^*^	0.31	0.14	0.20	0.18
	2. FACES BE	−0.12	1	0.49^*^	0.84	0.22	0.46^*^	0.37
	3. FACES MS	0.17	−0.22	1	0.88^**^	0.35	0.42	0.52^*^
	4. FACES Total	0.04	0.62^**^	0.61^**^	1	0.35	0.51^*^	0.52^*^
	5. Day-Night	0.46	−0.10	−0.28	−0.31	1	0.44	0.62^*^
	6. Digit Span FW	0.51^*^	0.06	0.21	0.22	−0.06	1	0.77^**^
	7. Digit Span BW	0.16	0.26	−0.23	0.22	0.14	0.2	1

False belief, FACES BE, Basic Emotion recognition, FACES MS Mental States, FACES Total, Day-night, Day and Night Stroop, Digit Span Forward, Digit Span Backward

** *p < 0.001*,

* *p < 0.05*.

### Behavioral questionnaire

**Figure 3:**
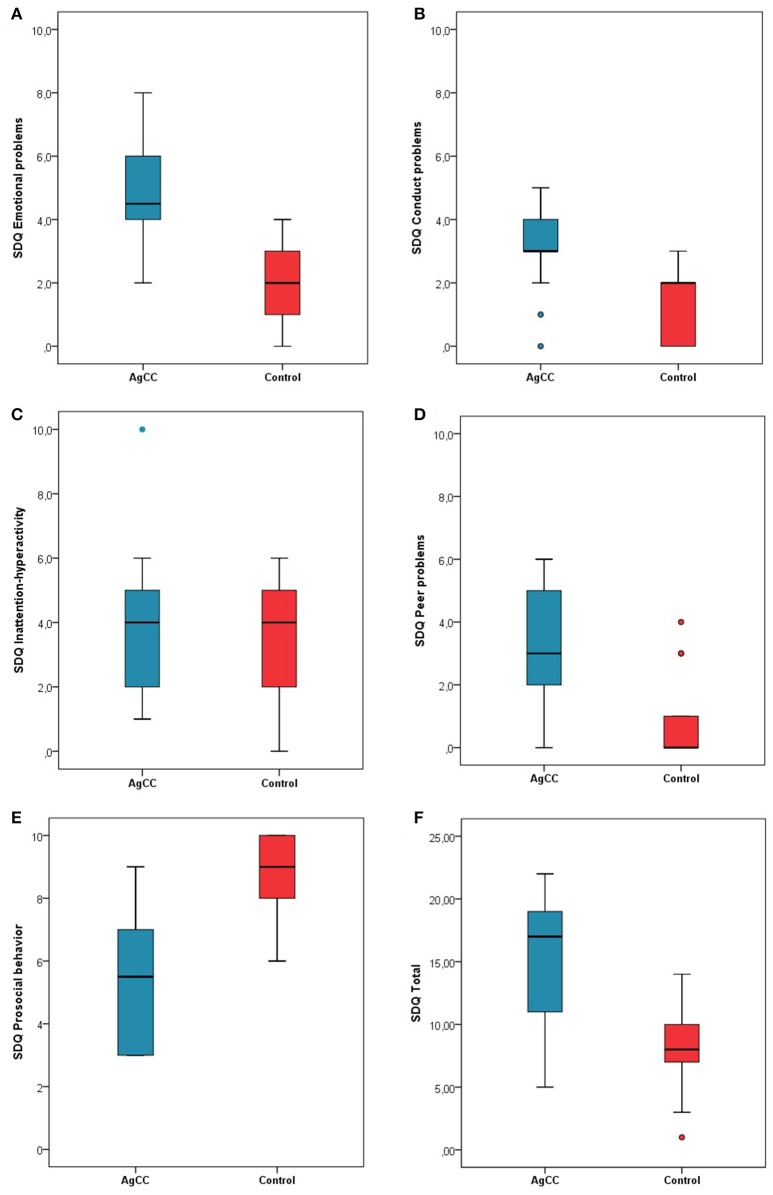
**Parent rating on Strength and Difficulties Questionnaire**. Mean scores for parent rating scales including **(A)** Emotional problems, **(B)** Conduct problems, **(C)** Inattention-Hyperactivity, **(D)** Peer problems, **(E)** Prosocial behavior, and **(F)** Total difficulties. Higher scores indicate greater symptomatology. Error bars represent standard deviation.

We compared differences in behavioral, emotional and relationship problems between the two groups using independent sample *t*-test, and computed Cohen's *d* effect size. Comparison of the SDQ subscale scores and the total difficulties revealed that children with AgCC had significantly higher mean scores (*p* < 0.001) on the total difficulties scale, and on all subscales, expect for the inattention-hyperactivity subscale (*p* = *ns*.). Table 5 shows the mean scores and Cohen's *d* effect size values in the AgCC cohort (Figure 3).

**Table 5:** **Results of Strengths and Difficulties Questionnaire (SDQ)**.

**(Sub)scales**	**AgCC (*N* = 18) Mean (*SD*)**	**Control (*N* = 18) Mean (*SD)***	**Effect size (Cohen's *d*)**	***t*-test**
Emotional problems	4.61 (1.50)	2.00 (1.23)	1.90	5.69^**^
Conduct problems	3.27 (1.31)	1.50 (1.15)	1.43	4.38^**^
Inattention-hyperactivity	4.00 (2.27)	3.72 (1.77)	0.13	0.40
Peer problems	3.16 (1.88)	1.88 (1.28)	0.79	4.64^**^
Prosocial behavior	5.47 (2.03)	8.69 (1.46)	1.81	−6.52^**^
Total difficulties	15.05 (5.17)	7.88 (3.17)	1.67	5.00^**^

*Number of children (N), SDQ mean scores, standard deviations (SD) and effect sizes for SDQ (sub) scales and t-test values*.

Statistical significance of differences between children with and without AgCC (t-test):

** *p < 0.001*.

### Relationships between social-cognitive factors and behavioral problems

We carried out further correlational analysis for each group separately to determine correlations between social and cognitive factors, and the severity of behavioral problems. The analysis involved the following factors: ToM ability (the total score of false belief tasks), emotion and mental state recognition (Faces: basic emotion recognition, complex mental states recognition, and Faces total score), and executive function (inhibitory control: Day and Night Stroop incongruent condition, working memory: digit span forward and digit span backward). The relationship of social cognitive factors to behavioral problems was examined separately for each SDQ subscale (Table 6). The AgCC group revealed a borderline significant correlation between ToM and the SDQ Peer Problems subscale [*r*_(17)_ = −0.45, *p* = 0.06], and the Prosocial subscale marginally correlated with ToM [*r*_(17)_ = 0.46, *p* = 0.06], complex mental state recognition [*r*_(18)_ = 0.42, *p* = 0.08], and inhibitory control [*r*_(15)_ = 0.48, *p* = 0.06]. In the control group, a significant correlation was found between complex mental state and the SDQ Inattention-hyperactivity subscale [*r*_(17)_ = −0.51, *p* = 0.03], and digit span backward and SDQ Conduct problems subscale [*r*_(17)_ = −0.49, *p* = 0.03].

**Table 6:** **Correlation *r* between social factors and behavioral problems measures for the whole sample and for separately for each group (AgCC Group/Control Group)**.

	**SDQ**					
	**Emotional problems**	**Conduct problems**	**Inattention-hyperactivity**	**Peer problems**	**Prosocial behavior**	**Total difficulties**
False Belief	−0.47^**^ (−0.22/−0.23)	−0.37 (−0.11/−0.08)	−0.03 (0.22/−0.31)	−0.55^**^ (−0.45/−0.24)	0.57^**^ (0.48/0.34)	−0.47^*^ (−0.17/−0.39)
FACES BE	−0.26 (−0.04/0.01)	−0.25 (−0.08/−0.02)	−0.05 (−0.09/0.09)	−0.32^*^ (−0.07/−0.30)	0.29 (0.16/−0.09)	−0.16 (−0.14/−0.08)
FACES SE	−0.36^**^ (0.35/−0.13)	−0.12 (0.27/0.22)	−0.06 (0.16/−0.51^*^)	−0.40^**^ (−0.19/0.01)	0.58^**^ (0.43/0.23)	−0.33^*^ (0.81/−0.25)
FACES Total	−0.36^**^ (−0.02/0.11)	−0.21 (0.12/0.15)	−0.01 (0.05/−0.33)	−0.44^**^ (−0.16/−0.23)	0.52^**^ (0.34/0.11)	−0.34^**^ (−0.07/−0.27)
Day-Night	−0.39^*^ (−0.36/0.01)	−0.47^**^ (−0.18/−0.02)	−0.01 (−0.08/0.28)	−0.46^**^ (−0.18/0.11)	0.71^**^ (0.48/0.19)	−0.43^*^ (−0.05/0.20)
Digit Span FW	−0.04 (−0.24/0.08)	−0.13 (−0.28/0.08)	−0.17 (−0.15/−0.30)	−0.05 (−0.26/0.01)	−0.08 (−0.03/0.04)	−0.09 (−0.21/−0.25)
Digit Span BW	−0.22 (−0.31/−0.05)	−0.13 (−0.07/−0.49^*^)	−0.05 (−0.22/0.17)	−0.05 (−0.03/0.04)	0.21 (0.06/0.22)	−0.16 (0.14/0.14)

False belief, FACES BE, Basic Emotion recognition, FACES SE, Social emotion recognition, FACES Total, Day-night, Day and Night Stroop, Digit Span Forward, Digit Span Backward

** *p < 0.001*,

* *p < 0.05*.

We used a value of *z* (Weaver and Wuensch, 2013) that can be applied to assess the difference between two correlation coefficients of the AgCC group and control group. We computed *z*-tests for each pair of correlations. The *z*-test results showed that the observed correlations did not differ from one another between two groups (*z* coefficients ranged from *z* = −1.08 to *z* = 1.66, *p* = ns.), except for the relationship between Hyperactivity-inattention subscale and complex mental state recognition (*z* = −1.98 *p* = 0.02). This finding indicates that the relationship between social cognition and the severity of behavioral problems in children with and without AgCC does not differ significantly. This means that typically developing children and AgCC children represent the two endpoints of the same scale. Control children showed fewer behavioral difficulties, with good performance in social and cognitive tasks, while AgCC children showed more behavioral difficulties associated with weaker cognitive and social abilities.

## Discussion

In order to provide evidence to clarify the role of the corpus callosum, regarding the nature of understanding others' mind, we investigated the main socio-emotional and cognitive functions in a group of children with isolated AgCC. We administered theory of mind tasks, emotion/mental state recognition, executive measures, and a parent-reported behavioral problems questionnaire. The findings of the present study are in line with previous studies showing typical mild social cognitive impairments in individuals with AgCC, even in childhood.

On the theory of mind tasks, children with AgCC performed significantly poorer than age- and IQ-matched controls. AgCC children performed poorly on both false belief tasks, with only half of the AgCC children passing the first-order-false belief task compared with 89 percent of control children. Only 23% of AgCC children passed the second-order false belief task compared with 55% of normally developing children in our sample. These findings suggest that children with AgCC have an increased risk of having problems in understanding other people's mind. Similar deficits in theory of mind are known from findings in children with autism. They typically fail the false belief task (e.g., Smarties Tube Task or Sally Anne task), while 4-year-old normally developing children, or even children with Down syndrome, are able to pass it. A previous study with AgCC adults (Symington et al., 2010) also found mild theory of mind deficits in various mentalizing tasks, such as understanding sarcasm and interpreting visual and textual social cues. However, this study has not reported serious deficits in specific theory of mind tasks that required mental state attribution (Faux Pas Test and Happé Theory of Mind Stories). A more recent study (Paul et al., 2014), directly comparing the social functions in an AgCC group and an ASD group, reported that AgCC adults had higher empathizing scores than ASD adults. These findings together indicate that individuals with AgCC have difficulties in understanding others' mental state, and they share some impaired social cognition with ASD persons, but AgCC individuals seem to have better mentalizing capacities. Our findings support this conclusion; children between the age of 6 and 8 years with callosal dysgenesis also indicated developmental delay in standard ToM tasks, but their performance showed high variability, ranging from severely impaired to “perfectly normal,” and their ToM performance was not associated with intelligence factors or any executive functions.

With respect to emotional and mental state understanding (Faces Test, Baron-Cohen et al., 1997), children with AgCC had fewer difficulties recognizing basic emotions from photographed facial expressions; they only showed some deficits on negative emotion trials (angry-fear and disgust-sad distinctions). In contrast, the AgCC children showed more deficits in recognizing complex mental states compared to the control children. In particular, AgCC children failed to identify expressions that depicted a specific mental state (interest vs. disinterest), or a complex social emotion (scheming vs. arrogant, and guilt vs. arrogant). Similar impaired recognition of emotions has been shown in previous studies, which examined social-emotional abilities in clinical samples. Individuals with schizophrenia have difficulties recognizing emotion expression of disgust (Bediou et al., 2005), anger (Gogharie and Sponheim, 2013), fear, and surprise (Barkl et al., 2014). While individuals with autism often fail to recognize fear and anger, they tend to mislabel anger as fear (Pelphrey et al., 2002). Additionally, the impaired recognition of complex mental states is more well-known in subjects with autism (Baron-Cohen et al., 1997). A prior study with AgCC subjects (Bridgman et al., 2014) found similar patterns of emotion recognition. AgCC individuals also had difficulties in recognizing fearful expressions and they often mislabelled anger as disgust or sadness, and fear as surprise. Individuals with callosal dygenesis also showed atypical face perception, including reduced gaze to the eyes and increased focus on the mouth. In line with this evidence, the present findings indicate that children with AgCC are able to identify most of the primary emotions, however, AgCC children have some difficulties in detecting a complex mental state from facial expressions, particularly those expressions that require processing information from the eyes (e.g., interest, guilt, arrogance, scheming). This finding supports the idea that AgCC and ADS individuals share some impairment in social cognition.

The executive functions in AgCC children showed a normal range of working memory abilities, but difficulties in performance of inhibitory control; the Stroop-like interference was higher than the performance of control children. Executive functions are a set of higher-order cognitive skills that depend upon specific callosal connectivity. A comprehensive study (Marco et al., 2012) directly investigating the executive functions in an AgCC cohort also found impairments in inhibition and flexibility tasks, but the performance in executive tests was attributed to slow cognitive processing. In contrast, Brown et al. (2001) found evidence that individuals with AgCC have normal executive functions with respect to the inhibition/flexibility skills. They speculated that the presence of other cerebral commissures in AgCC allow for the interhemispheric transfer of information in inhibitory control tasks. The inconsistency of findings, probably comes from the high variability of difficulty levels in test batteries that were used. It is likely that AgCC individuals exhibit more errors and slower processing speed when the information is more complex and less easily encoded. Our findings also showed that children without the corpus callosum committed more errors in interference tasks. Response time was not measured reliably in our study; therefore, we have no information on whether this performance is a consequence of processing speed when the nervous system uses the alternative routes connecting the hemispheres.

Taken together, findings presented here support several potential explanations that may account for the impaired social cognition in children with AgCC. First, the language explanation suggests that the impaired social function is mediated by the decreased capacity in linguistic pragmatics. Previous findings of linguistic studies have also suggested that AgCC patients tend to use the literal meaning of the narrative information and ignore the second-order meaning of narratives or conversations. It is possible that children with AgCC have trouble understanding the false belief tasks because they do not understand different perspectives in the context of ToM tasks required to describe the world linguistically. Additionally, AgCC individuals tend to use fewer “mentalizing words” (Symington et al., 2010), that reflect others' mental states (“know,” “think,” “feel”), resulting in deficits that infer mental and emotional processes of others. The lack of callosal interconnectivity may support the decreased capacity in linguistic pragmatics, as the callosal dysgenesis reduces the accessibility to the more complex integration of the semantic network, which is widespread in the two hemispheres. According to the second explanation, executive functioning is also a potential candidate for mediating impaired social cognition in AgCC patients. The absence of callosal connections in AgCC functional brain connectivity seems to be more limited during tasks that require complex cognitive operation, such as inhibitory control, working memory, and flexible switching. A previous brain imaging study (Hinkley et al., 2012) demonstrated that impairment of functional interaction appears in regions in the frontal, parietal, and occipital cortices, which indicated social-cognitive functions, known to be impaired in AgCC patients. Indeed, performance in executive function (Tower of London task) directly correlated with resting-state functional connectivity of dorsolateral pre-frontal cortex in individuals with AgCC (Hinkley et al., 2012). Our findings, however, do not support that executive functions, namely the inhibitory control, reflect the impaired social cognitive function, like theory of mind and mental state recognition. A third possible explanation is the deficit in the process of integration of multiple sources of information. AgCC patients have difficulties using the context of a complex situation to interpret the meaning of linguistic information, and inferring the mental states of other persons based on the available, simpler information. The absence of the corpus callosum disrupts the interhemispheric connection and limits the size of the functional processing network of complex social cognitive functions. However, individuals with complete AgCC do not experience disconnection syndrome, they exhibit a limited amount of interhemispheric transfers, mediated by anterior commissure and additional alternative anatomical tracts, developed in AgCC subjects, such as Probst and heterotopic bundles, providing compensatory mechanisms for social cognitive processes (Marco et al., 2012). However, these alternative connections cannot compensate fully for the complex function of the corpus callosum, and results in a slower processing speed. Moreover, the lacking interhemispheric connection leads to an alteration in intrahemispheric connectivity that increases the likelihood of other cognitive deficits. Other developmental disorders, such as ASD, ADHD, and schizophrenia also demonstrated reduced callosal size that contributed to impairments in interhemispheric transfer and processing speed (Paul et al., 2007).

The secondary aim of this study was to investigate whether difficulties in social cognition and/or executive functioning are related to the severity of behavioral problems, and whether they increase the prevalence of emotional- and social problems in children with AgCC. Mental health problems were assessed by the Strength and Difficulties Questionnaire, which had never been used before in a sample of children with AgCC. Using parent-administered questionnaires, we found that children with AgCC had more problems with all domains (behavior, emotions, prosocial behavior, and relationship), except the inattention-hyperactivity domain, compared with control subjects. However, the lack of differences in the Inattention-Hyperactivity subscale can be explained by the fact that there were three children in the control group who also reached the cut-off score on the Inattention-Hyperactivity subscale. The results of the SDQ indicate that the functional changes in brain connectivity might contribute to behavioral problems in childhood, as several previous studies reported mild to severe behavioral anomalies in AgCC individuals, with the most frequently mentioned disorders being autism and attention deficit hyperactivity disorder (ADHD) (Paul et al., 2007). The abnormal development of connectivity during childhood is likely to mediate the reduced capacity in complex social cognitive processes, which may contribute to the symptoms of behavioral problems or early onset of psychiatric disorders. The findings within the AgCC group demonstrated that the deficits in social cognitive skills are only marginally correlated with behavioral problems. AgCC children show mild dysfunction in three domains of mental state recognition, theory of mind, and executive function (inhibitory control), and these dysfunctions are associated with behavioral problems. It seems that the peer problems and prosocial behaviors are linked to mentalizing capacity (false belief and complex mental state) and inhibitory control, and the complemental state recognition and inhibitory control are related to the Prosocial behavior subscale. Children who lack the capacity for understanding others' mind have more problems in social domains.

One of the limitations of this study the relatively small size of sample, which might have prevented findings the hypothesized correlation between the socio-cognitive factors the behavioral adjustment. Another limitation of our findings are that the AgCC cohort involved children with partial corpus callosum agenesis (pAgCC), whose behavioral performance did not differ from the complete AgCC, but the different brain condition might predict variable social-cognitive performance. Previous studies showed that the residual fibers of the callosum of pAgCC probably provide higher variability in the pattern of interhemispheric connectivity (Wahl et al., 2009), increasing the variability of the behavioral and cognitive outcome. Further study is necessary in order to understand how the compensatory anatomical changes and residual callosal tracts contribute to the social and cognitive functioning. It may be more informative to investigate the emerging social cognitive performance and mapping of the developmental tracts assessed with MR and DTi imaging techniques, in parallel.

In conclusion, this study demonstrated that there are mild deficits in mental state understanding, executive functions, and behavior symptoms in children with AgCC. The findings indicate that dysgenesis of the corpus callosum constitutes a specific risk factor for developing social cognitive symptoms. AgCC individuals tend to misconstrue social information and misunderstand the mental states of others within complex social contexts, including problems with emotion recognition and complex mental state recognition, theory of mind, and inhibitory control. The absence of the corpus callosum seems to affect the development of behavioral characteristics and cause specific behavioral anomalies. Taken together, evidence from previous studies with AgCC patients suggest that social cognitive impairments may relate to the missing corpus callosum. The callosal agenesis results in deficiencies in imagining and inferring the mental, emotional, and social functioning of others. This pattern of cognitive and social deficits has been labeled as *primary AgCC syndrome*, by Symington et al. (2010) for that condition, when there is callosal absence without evidence of other brain pathology or the observable cognitive and social impairments *primarily* related to the absence of the corpus callosum. The primary AgCC syndrome profile includes impaired emotion recognition, restricted verbal interpretation of social scenes, and emotional experiences, as well as mild deficits in theory of mind. The lack of callosal interconnectivity might explain the decreased capacity in the higher-order cognitive domain, as the callosal dysgenesis reduces the accessibility to the more complex integration of social networks, which is widespread in the two hemispheres. In AgCC individuals it is likely that the functions involved are those that are hemispherically lateralized (emotions, language, visuospatial processing), or the complex social functions, in which the information is spatially distributed between the two hemispheres. If the development of the corpus callosum is impaired, the normal interaction and competition between the hemispheres is abolished, resulting an alternative routes in the adult brain.

## Informed consent

Written informed consent was obtained from patients who participated in this study.

## Author contributions

BL as an author contributed to the following tasks during the preparation of this manuscript: planning research method, collecting and analyzing data, writing the manuscript. AB as an author contributed to the following tasks during the preparation of this manuscript: planning research, examining children concerning the medical conditions, analyzing data and writing manuscript.

### Conflict of interest statement

The authors declare that the research was conducted in the absence of any commercial or financial relationships that could be construed as a potential conflict of interest.
